# Patterns of Population Differentiation and Natural Selection on the Celiac Disease Background Risk Network

**DOI:** 10.1371/journal.pone.0070564

**Published:** 2013-07-31

**Authors:** Aaron Sams, John Hawks

**Affiliations:** 1 Department of Anthropology, University of Wisconsin-Madison, Madison, Wisconsin, United States of America; 2 Department of Biological Statistics and Computational Biology, Cornell University, Ithaca, New York, United States of America; Centro di Riferimento Oncologico, IRCCS National Cancer Institute, Italy

## Abstract

Celiac disease is a common small intestinal inflammatory condition induced by wheat gluten and related proteins from rye and barley. Left untreated, the clinical presentation of CD can include failure to thrive, malnutrition, and distension in juveniles. The disease can additionally lead to vitamin deficiencies, anemia, and osteoporosis. Therefore, CD potentially negatively affected fitness in past populations utilizing wheat, barley, and rye. Previous analyses of CD risk variants have uncovered evidence for positive selection on some of these loci. These studies also suggest the possibility that risk for common autoimmune conditions such as CD may be the result of positive selection on immune related loci in the genome to fight infection. Under this evolutionary scenario, disease phenotypes may be a trade-off from positive selection on immunity. If this hypothesis is generally true, we can expect to find a signal of natural selection when we survey across the network of loci known to influence CD risk. This study examines the non-HLA autosomal network of gene loci associated with CD risk in Europe. We reject the null hypothesis of neutrality on this network of CD risk loci. Additionally, we can localize evidence of selection in time and space by adding information from the genome of the Tyrolean Iceman. While we can show significant differentiation between continental regions across the CD network, the pattern of evidence is not consistent with primarily recent (Holocene) selection across this network in Europe. Further localization of ancient selection on this network may illuminate the ecological pressures acting on the immune system during this critically interesting phase of our evolution.

## Introduction

Celiac disease (CD) is a common, highly heritable [1], small intestinal inflammatory condition induced by wheat gluten and related proteins from rye and barley [2]. Specific risk alleles of the HLA-DQA1 and DQB1 genes encoding the DQ2 and DQ8 heterodimers appear to be necessary, but not solely responsible for development of CD [3]. These specific HLA genotypes explain approximately 40 percent of the genetic risk for CD. HLA-DQ heterodimers encoded by these risk alleles present cereal peptides to CD4+ T cells, activating an inflammatory immune response in the small intestine [4]. Recent genome-wide association studies (GWAS) and linkage analyses have identified at least forty non-HLA loci, many of which lie in genomic regions that earlier work had already associated with immune function [5]–[10]. Unlike the case of HLA, the exact causal mechanisms for these other CD risk associations are mostly unknown.

Bioarchaeological evidence shows that dietary and demographic transitions of the Holocene were detrimental to human health [11]. Dietary specialization led to nutritional deficits and denser living conditions created ideal conditions for the spread of new pathogens, as documented by higher rates of porotic hyperostosis, cribra orbitalia, dental caries, linear enamel hypoplasias, tuberculosis, and trepanematoses in populations after the onset of agricultural production [11], [12]. Genetic and archaeological evidence show the importance of adaptive evolution concurrent with large-scale demographic change over the last 10,000 years [13]. These observations provoke the hypothesis that the Holocene transition to agriculture was a time of strong natural selection on genes important to diet and immunity.

CD appears to be an evolutionary paradox. It is common today (>1%) in several populations with long histories of wheat agriculture including Europe and the Near East. The effectiveness of a gluten-free diet as a treatment has been clinically recognized only since the discovery of gluten as the CD trigger in the 1950’s [14]. In past populations CD should have had a high potential to reduce the fitness of its sufferers directly, or by interfering with nutrient absorption, or by other effects that reduce fertility [15]. The idea that CD would have reduced past fitness was recently bolstered by skeletal and DNA evidence from a roughly 2,000 year-old female from the archaeological site of Cosa, near Tuscany, Italy. This skeleton includes classic paleopathological signs of infection and malnutrition leading to death prior to age 20 and also contains the most common HLA-DQ risk haplotype (DQ2.5) for CD [16]. A long history of CD risk in European and Near Eastern populations requires some evolutionary explanation. Founder effects in ancient populations might have increased the frequency of deleterious CD risk alleles, or these alleles may have been influenced by positive selection on their other pleiotropic effects. Much evidence supports the role of recent natural selection in shaping the HLA region [17]. Less well understood is the influence of selection on the more than 40 non-HLA loci associated with CD risk.

One study has suggested strong recent selection on some of these GWAS CD risk loci. Zhernakova and colleagues [5] found evidence of recent selection in or around the genes IL12A, IL18RAP, and SH2B3. The risk variant in the SH2B3 gene is functionally involved in the NOD2 recognition pathway, suggesting that it may have been positively selected to protect against bacterial infection. The authors inferred a very recent onset of selection (between 1,200 and 1,700 years ago) by extended haplotype homozygosity (EHH) [18].

We investigated whether the signals on these three loci are consistent with the broader genetic network influencing CD risk. A null hypothesis is that CD-associated gene loci have undergone the same dynamics of demography and selection as the rest of the genome. We tested this hypothesis by evaluating the differentiation of the chromosomal regions flanking CD-associated loci, both between and within continental populations. Owing to the spread of humans around the globe and their subsequent rapid population growth, evidence of recent selection tends to be geographically localized within continents or smaller geographic regions [13], [19]. For this reason, gene networks whose components have been subject to recent selection may show significant regional differentiation when compared to loci randomly drawn from across the genome [20]. In the case of CD selection on standing variants may have been just as important as selection on new mutations and a test across many loci may find evidence missed in the examination of single loci with linkage disequilibrium (LD) based approaches. GWAS risk loci are not necessarily causal variants, so we tested flanking regions for evidence of population differentiation. By comparing a hierarchical set of populations both within continents and between continents, we investigated the time and geographic distribution of adaptive variation in this gene network.

## Results

To investigate broad-scale geographic differentiation between continental regions we assessed the fraction of SNPs in each CD risk locus that fall in the top 1% of the genome-wide F_ST_ distribution (assessed from over 2 million randomly chosen SNPs) for that between-continent comparison. Further, that fraction of high F_ST_ SNPs at each locus was assigned a significance (P) value based on a genome-wide random sample of 11,000 loci.

The regions around CD risk loci are much more likely to show significant intercontinental differentiation than are SNP loci randomly chosen across the genome. Averaged over all CD loci, the proportion of high F_ST_ outliers exceeded 1% in all three between-continent comparisons (Table S1). In our bootstrapping procedure, thirteen of fifty-four (∼24%) of the regions flanking independent CD risk loci are significantly differentiated (have more than expected high- F_ST_ SNPs, P<0.05) for at least one between-continent comparison. This may be a slight overestimate due to the fact that flanking regions overlap in a few loci.

**Figure 1:**
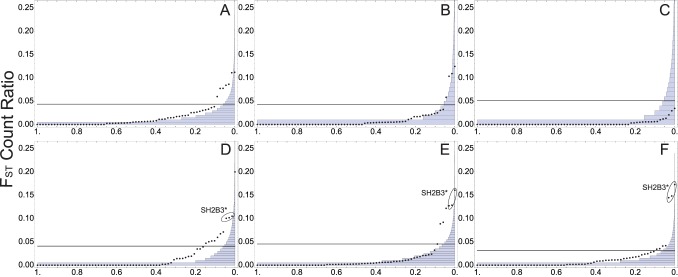
Genome-wide high-F_ST_ ratio decay and non-HLA risk loci values. In each plot, the histogram represents the genome-wide distribution (11,000 samples) of the fraction of SNPs in each locus with F_ST_ higher than the 99% genome-wide upper boundary (black line) this distribution is plotted against the high- F_ST_ ratio for each CD risk locus bin in this analysis ordered from smallest to largest. Dots above the line represent loci with significantly elevated high- F_ST_ ratios (P<0.05). Raw data for CD regions is found in Table S1. a. within Africa, b. within East Asia, c. within Europe, d. between Europe/East Asia, e. between Africa/East Asia, f. between Africa/Europe.

The differentiation of the CD risk network stands out most prominently from other loci when comparing European and Asian samples (Figure 1). In this case, the simplest comparison is the overall fraction of high- F_ST_ SNPs across all regions. For Europe and Asia, 1.5 percent of SNPs across the regions flanking CD risk loci exceed the 1 percent genome-wide threshold (Table S1). In other words, fifty percent more high- F_ST_ SNPs are found in these regions than expected from the genome-wide distribution. These SNPs are linked into regions and so the appropriate significance test on the network is based on the proportion of regions with an excess of high F_ST_ SNPs. High F_ST_ SNP loci are especially clustered within nine of the chromosomal regions around CD risk-associated loci. Nine of these regions are significant in a Europe-Asia pairwise comparison genome-wide at P<0.05, two of which are significant at P<0.01 (Figure 1). This high proportion of high F_ST_ blocks is strongly significant (P<0.01) when considering the total number of comparisons.

The Europe-Africa comparison also shows a highly elevated fraction of high F_ST_ SNPs. However, these are clustered mainly in four of the chromosomal regions (Figure 1). The number of regions showing elevated F_ST_ between Europe and Africa is therefore not significant. The four high F_ST_ regions include two loci that show significant evidence of selection by long-range linkage [5], [21], [22]. To the extent that these loci do show evidence of selection, our comparison across the network does not show that other loci have been significantly selected in the history of European and African population differentiation.

We consider this pattern of between-continent results a rejection of the null hypothesis (H_0_) that the CD background risk network has experienced the same pattern of evolution compared to the genome as a whole. The results point in an interesting direction. Loci identified by earlier long-range haplotype tests as recently selected (such as the SH2B3 locus on chr 12) account for a high fraction of the high- F_ST_ SNPs in all comparisons. Furthermore, evidence of elevated F_ST_ across the broader CD network that has not been shown to be subject to positive selection is concentrated between Europe and East Asia. Importantly, the loci with elevated F_ST_ between Europe and East Asia are, for the most part, not the same loci that are highly differentiated between Europe and Africa. Therefore, we may be picking up a signal of selection on standing variation, not strong positive selection, in addition to previously demonstrated evidence of recent positive selection on two of these loci. Evolution of the CD risk network within Europe might account for some of these observations.

We turned to within-continent comparisons to address whether evolution of the CD risk-associated loci occurred uniformly over time. Previous work suggested that a proportion of autoimmune genetic risk (including CD) may reflect positive selection on the immune system within the last 10,000 years [5], [21]–[23]. If the selection that we identified with between-continent comparisons were very recent, we might expect additional loci to show high- F_ST_ fractions. If there were more recent (Holocene) selection in Europe or East Asia, we might expect additional loci to show up as significant outliers when comparing populations within each continental region. What we observe is the opposite. Within Africa, approximately five independent regions show a significant excess of high- F_ST_ SNPs. Within Asia, only four regions have a significant excess of high- F_ST_ SNPs. In Europe there is a strong deficit of high- F_ST_ SNPs. No within-continent comparisons show a significant (P<0.05) excess of regions with a significant (P<0.05) ratio of high- F_ST_ SNPs.

This result may appear to contradict previous evidence of strong recent selection on at least four of these loci [5], [21], [22]. The contradiction might be a unique property of the evolution of these loci for which our comparisons, directed to the broader issue of selection across the entire network, may be less informative. We chose to investigate one of these loci further. The Iceman genome [24] provides an alternative test of recent selection on the CD risk network. The strongest prior evidence of selection in this network is the risk variant rs3184504 in SH2B3 reported by Zhernakova and colleagues [5]. This locus is represented in the data from the 5,300-year-old Ötzi genome. Zhernakova and colleagues estimated an age for this locus using the EHH statistic of only 1,200–1,700 years ago. This age estimate makes the clear prediction that the iceman genome should not have the risk allele. However, Ötzi is a heterozygote carrying this allele. For this reason, we propose that the evidence of selection on this locus may actually pertain to a time period well before 5,300 years ago.

**Figure 2:**
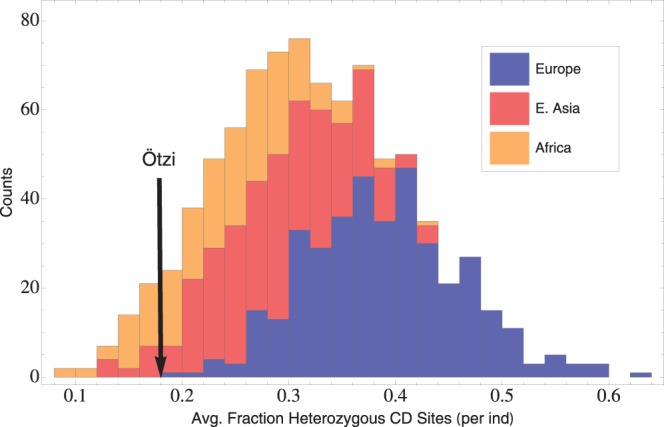
Mean observed heterozygosity across CD GWAS SNPs from 1000 Genomes and Iceman. Histograms represent the distributions of observed average heterozygosities across 49 GWAS SNPs associated with CD in Europeans (Blue), East Asians (Red), Africans (Orange), and the Iceman (black arrow). This figure illustrates that the Iceman’s mean heterozygosity across CD associated loci is low compared to present-day Europeans.

**Figure 3:**
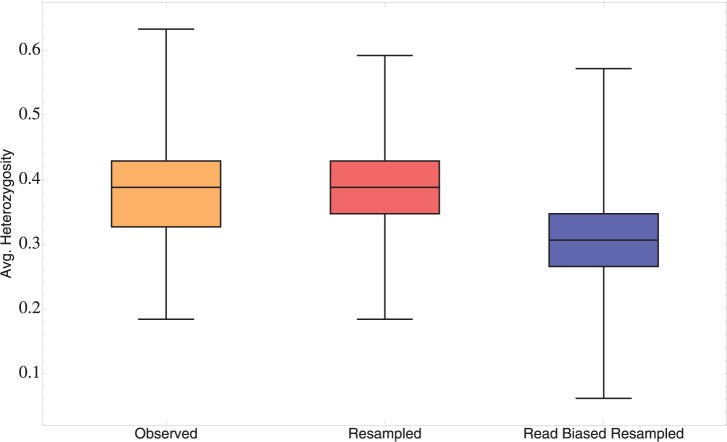
Estimated effect of low coverage in the Iceman genome on mean heterozygosity at GWAS loci. Box plot illustrating the distribution of observed mean heterozygosity across 49 GWAS SNPs in present Europeans from the 1000 Genomes Project (Orange), the expected distribution of mean heterozygosity based on resampling from allele frequencies in Europe (Red), and the expected distribution of mean heterozygosity based on allele frequencies in Europe and random sampling of alleles according to the distribution of read coverage in the Iceman (Blue). Although there is a significant reduction in mean heterozygosity, the range of values is similar, suggesting that the observed reduction of heterozygosity in the Iceman compared to present Europeans is genuine.

We do not know the extent to which GWAS SNPs contribute to CD risk, but the Iceman genotypes can provide a rough test of positive selection across the network even without this information. The Ötzi genotype was drawn from the European population of 5,000 years ago. If the ancient population was different from today’s European population in the frequencies of SNP alleles associated with CD risk, then Ötzi will carry some genotypes that may be unlikely given their frequencies today in Europe. Ötzi is a heterozygote at nine out of forty-nine total GWAS risk sites (mean heterozygosity ≈ 0.184). The average heterozygosity in the Ötzi genome across these loci is identical to the minimum heterozygosity among Europeans in the 1000 Genomes Project sample today and closest to the mean heterozygosity observed in Africa (Figure 2). Low coverage in the Ötzi genome could lead to a bias in the ascertainment of heterozygous sites due to chance. We tested whether a coverage bias could explain the reduction in heterozygosity across the GWAS risk sites (avg. coverage = 5.6) by randomly generating sets of genotypes based on allele frequencies in modern Europeans and used these sets of genotypes to resample reads from each genotype randomly based on read number in each risk site in Ötzi. The resulting distribution of over one million randomly generated average heterozygosities demonstrates a marked reduction in ascertainment of heterozygous sites (Figure 3). Nonetheless, the average number of heterozygous sites that we measure for Ötzi remains in the bottom of the heterozygosity distribution for Europe. Because Ötzi is an outlier compared to present-day Europeans for genotypes across these CD risk loci, we cannot reject the hypothesis that strong positive selection has affected the frequencies of any large proportion of these loci during the past 5,000 years.

**Figure 4:**
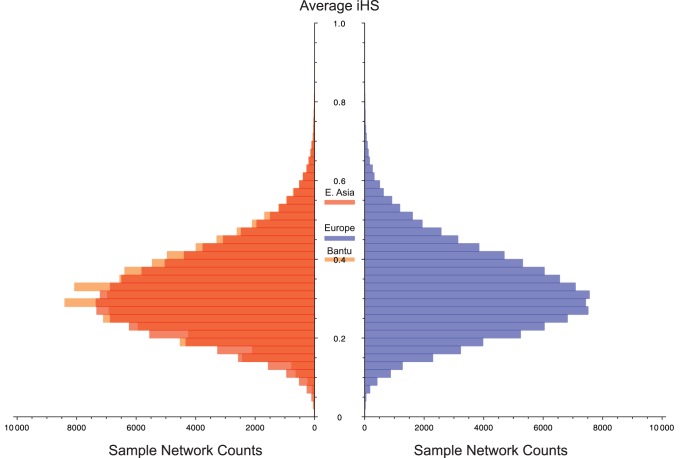
Within-continent mean normalized iHS values in CD network compared to genome-wide sample. Paired histogram illustrating mean iHS values of 100,000 sample networks of similar size to the CD network for European (Blue), East Asian (Red), and Bantu (Orange) samples from HGDP. Mean iHS values for the CD network in each region are indicated in the central margin of the histogram. Only the average value for East Asian samples is significant (p = 0.031). Networks were sampled requiring a minimum physical distance of 2 Mb between any two loci on the same chromosome.

As a separate means of assessing a signal of recent positive selection across the total network of CD risk loci, we utilized existing long-range haplotype data in the form of normalized integrated haplotype scores (iHS) [19], [25]. We performed a randomization test that measures the intensity of maximum iHS signals in the CD network compared to randomly generated sets of loci, genome-wide. The genome-wide distribution of randomly selected sets of loci include whatever fraction of loci in the genome actually was selected recently, therefore this test is conservative. This test was performed on European, East Asian, and Bantu iHS datasets downloaded from the UCSC genome table browser (see methods). Interestingly, there is no evidence of out of the ordinary mean iHS scores among the European (p = 0.114) or Bantu (p = 0.236) samples. In contrast, the East Asian sample p-value (0.031) is in the upper tail of the genome-wide distribution (Figure 4). These results suggest that the non-HLA autosomal network of genes underlying CD risk may have been under recent positive selection in East Asian populations.

## Discussion

Our within-continent comparisons show that the overall pattern of CD risk does not match the predictions of strong recent selection in Europe. Considered together, the CD risk loci had a higher than expected proportion of outlier SNPs in Africa and Asia, but not in Europe (Figure 1). Compared to the genome-wide value, the CD risk network showed the highest relative differentiation between Luhya (LWK) and Yoruba (YRI) samples. The two Chinese samples (CHB and CHS) are also more highly diverged than the genome-wide expectation. In contrast, the CEPH (CEU) and Tuscani (TSI) samples show a much smaller fraction of genomic high-F_ST_ outliers in the CD risk regions. Our intercontinental comparisons showed that selection likely differentiated East Asia and Europe at several CD risk loci, if that selection acted mostly early in the differentiation of Europe and East Asia, we could predict that European populations today might show low differentiation across this network. That is the result we observe. However, very recent selection on standing variation within Europe that acted in the same direction across this network in these European populations might also give rise to low differentiation of these populations as observed today. Therefore, the within-continent F_ST_ comparison by itself cannot rule out recent selection within Europe. Our randomization test on the CD risk network using iHS scores suggests that the CD network has not experienced an excessive amount of recent positive selection compared to the genome-wide average in Europeans and therefore, supports our F_ST_ result in Europe. Interestingly, this test suggests that the CD network may have experienced recent positive selection in East Asian populations (Figure 4). This signal may be contributing to the high F_ST_ values in our East Asia/European comparisons. It is important to recognize that the loci included in this study were identified by GWAS in European, not East Asian CD cohorts. Therefore, selection on this network in East Asian populations may have little to do with the natural history and etiology of CD. However, many studies suggest a general role for many of these risk loci in immune function and much overlap exists between CD risk and risk for other autoimmune diseases [5]–[10], [22], [26]. Follow up work should examine the history of this network in East Asian populations.

Recent genetic evidence from ancient mitochondrial and autosomal DNA [27]–[29] supports the hypothesis of large-scale demographic turnover in Europe associated with the spread of agriculture between 10,000 and 5,000 years ago. If Ötzi is not closely related to the ancestors of present Europeans, then a difference in genetic composition at GWAS sites may (perhaps more likely) reflect demography rather than selection. More refined resolution of the Neolithic transition will help to resolve this issue. These results are equivocal but illustrate both the value of and issues associated with using ancient DNA for testing hypotheses about the timing of recent genetic changes.

The goal of this study was to address the evolutionary paradox of celiac disease risk, by assessing whether non-HLA CD risk loci have been affected by selection in human prehistory. The HLA-DQ haplotypes that contribute most substantially to individual risk of CD are already strong candidates for selection due to their functional roles in immunity, but the functional interactions are less well understood for the wider background of loci associated with CD risk. Our results demonstrate that these loci, when considered as a network, also have been influenced by selection during the evolution of human populations. That selection increased the differentiation of CD risk loci between Europeans and other continental groups.

The linkage block that includes the SH2B3 locus identified by Zhernakova and colleagues [5] shows the strongest sign of selection in our analysis, in terms of differentiation between continents. Therefore, this locus is likely subject to increased selection compared to other CD associated risk loci. However, both its presence in these far-flung populations and the presence of the specific risk allele (rs3184504) in the 5,300-year-old Tyrolean Iceman show that it is likely much older than initially estimated by Zhernakova and colleagues.

The pattern at SH2B3 is not typical of the CD risk network as a whole, which shows significant but much weaker indications of past selection. The pattern of increased differentiation between continents is clear only when considering a relatively large number of CD risk loci and their flanking regions. Setting aside the large risk conferred by HLA-DQ CD risk alleles, the known genetic CD background risk seems to have been affected by a combination of evolutionary patterns. Many of the CD risk loci are consistent with the pattern of differentiation of the genome as a whole; a substantial (and significant) fraction have been subject to changes in frequency that increased differentiation between populations without creating the kind of linkage disequilibrium that triggers significant EHH or iHS tests for positive selection, while a handful of loci may have been affected by strong positive selection.

No individual loci are excessively differentiated within Europe and the genotypes of the 5,300-year-old Tyrolean Iceman potentially suggest a recent shift in allele frequencies at some CD risk loci. These observations lead to two alternatives. First, it is possible that there has been a great deal of recent selection across the CD network in Europe which has either been in the form of uniform selection on standing variation or positive selection in combination with recent demographic turnover in Europe. Alternatively, our between- and within-continent comparisons may simply represent selection earlier in the expansion of modern humans from Africa. This alternative is consistent with the lack of signal of selection found by other researchers at CD loci [5], [21], [22], as well as our own iHS test on the combined CD network. We can improve our resolution of the recent history of CD loci in Europe with either data from more European and Near Eastern populations or with more ancient DNA. Ötzi illustrates that our hierarchical framework of modern comparisons are lacking in resolution with respect to recent selection. Additionally, we still cannot rule out that recent selection in Asia and/or Africa primarily explains our between-continent results. The excess of differentiation between Europe and Asia that we observe in or F_ST_ test may be explained in large part by our observation of elevated iHS scores in the CD network in Asia. While it is beyond the purview of this paper, detailed population genetic analysis on higher coverage sequence data in diverse populations will help to reveal the extent to which selection affected loci in the CD background risk network.

Moving forward, additional analysis of CD risk loci utilizing a geographically broader sample of population data and perhaps more importantly a larger number of ancient DNA samples will be necessary to resolve the timing of selection across the CD network in Europe. That additional information will reveal the extent to which the cultural and demographic shifts associated with the spread of agriculture from the Near East influenced the current distribution of genetic variation at non-HLA CD risk loci.

## Methods

### 1000 Genomes Project data

We obtained variant calls for single nucleotide polymorphisms from the June 2011 data release of the 1000 Genomes Project (http://1000genomes.org) [30]. For our comparisons, we considered only the autosomes, chromosomes 1–22, and excluded the X chromosome from consideration. We utilized the subset of individuals from the following population samples: Utah (CEU), Finn (FIN), Spanish (IBS), British (GBR), Tuscan (TSI), North and South ethnic Han Chinese (CHB, CHS), Japanese (JPT), African ancestry in American Southwest (ASW), Luhya (LWK), and Yoruba (YRI).

### Celiac Regions

We utilized the list in Table 2 of Trynka and colleagues [7] of all autosomal genomic regions with a highly correlated genome-wide significant signal of celiac risk. In our F_ST_ analyses we chose the center of each independent locus and assessed F_ST_ in the region spanning 25 Kb upstream and downstream of this central position. For loci much longer than 50 Kb we broke the locus into non-overlapping 50 Kb intervals. The resulting regions were mapped to human genome build 19 (hg19) using the UCSC genome build liftover utility (genome.ucsc.edu). The original regions from Trynka and colleagues [7], nearby genes, our loci mapped to hg19, and the results of our F_ST_ comparisons can be found in Table S1. We utilize physical distance rather than recombination map distance primarily as a means of programming efficiency. To ensure that this choice did not bias our results, we assessed average recombination rates in the set of CD loci to ensure that they do not have systematically lower recombination rates than the genome-wide expectation and that low recombination rates are not correlated with elevated F_ST_ ratios (data not shown).

### Iceman Genome

We examined the recently published genome of the 5,300 year old Tyrolean Iceman (Ötzi) [24] to provide a further test of the hypothesis of recent selection across the CD risk network in Europe. We obtained aligned genome reads from the Ötzi from the European Short Read Archive. At the time of our download, the Ötzi data were provided as three BAM files; we used samtools (http://samtools.org) to merge these into a single dataset. This allowed us to examine the probability of heterozygosity at a sample of SNP sites in the catalog of Genome-Wide Association Studies (accessed 3 July 2012) [31] associated with CD risk. We extracted all unique CD risk sites for which data was available in the Iceman genome. The final sample included forty-nine SNP sites. Since the risk variant is not reported for all sites in the GWAS catalog, we considered average heterozygosity across all forty-nine sites as an estimate of the degree of background risk present in a single individual and compared the Iceman to all individuals in the 1000 Genomes Project dataset samples listed above.

### Sample Statistic

We calculated sample weighted F_ST_ for all comparisons using in-house Python scripts (www.python.org) according to the method outlined by Akey and colleagues [32].

Pairwise F_ST_ was calculated between each Old World continental region by combining all samples from each geographic region. Additionally, we assessed the differentiation within each region by comparing a single pair of populations from the region (LWK×YRI, CHB×CHS, CEU×TSI). For our within-continent comparisons we are interested primarily in differentiation since the initial separation of populations within the region. Different continental regions are represented by unequal numbers of populations with unknown demographic histories. Therefore, rather than calculate a weighted F_st_ average, we chose to restrict our within-continent comparisons to population pairs in order to maximize the strength of the F_ST_ signal within each region.

### Differentiation at Each Locus

We pursued an empirical approach similar to Pagani and colleagues [33]. To determine the level of differentiation at each CD risk locus we calculated pairwise F_ST_ for all sites in the region that were segregating in at least one sample in the comparison (F_ST_ >0). We used an empirical approach instead of a model-based approach because of the heterogeneity of human population histories and our lack of detailed knowledge about them across the entire timescale important to CD risk. Demography affects all loci across the genome, so a demonstration that a group of loci is surprisingly different from the genome-wide pattern of differentiation would allow us to reject the hypothesis that demography alone is sufficient to explain their distribution. The 99% upper boundary for a randomly chosen set of over 2 million (2,200,000) SNPs from the entire genome was used as the empirical significance level for each comparison. Therefore, we considered as outliers all SNPs exceeding this upper boundary. A region was considered excessively differentiated if more than 1% of SNPs in the region exceeded this upper boundary.

### Multiple Comparisons Correction

While the above approach is a first step to determine if a region has an elevated proportion of high F_ST_ variants, this method does not include a formal statistical test. Yu and colleagues [34] demonstrated the value of empirical comparison of a candidate locus to randomly selected regions of the genome ascertained in a similar manner to detect signs of natural selection. Such comparisons implicitly account for the problem of multiple testing. Therefore, to determine the significance of each CD locus, for each population comparison we randomly selected eleven thousand 50 Kb regions from the genome and calculated the fraction of excessively differentiated SNPs for each of these (using the same process as for the CD loci). The number of randomly selected genomic regions with a fraction of excessively differentiated SNPs greater than or equal to that of the CD locus was taken as an empirical P-value for the CD locus. We are interested in whether or not each locus is excessively differentiated relative to the genome-wide average. Therefore, our final 5% significance level does not require additional adjustment. All comparisons were analyzed using in-house Python scripts (www.python.org).

Our test is a one-tailed comparison screening for an excess fraction of SNP loci with high F_ST_ across a region. Low- F_ST_ regions may also reflect interesting evolutionary dynamics, but we have chosen to test the hypothesis that regions have experienced the same evolutionary dynamics. This hypothesis can be rejected if directional selection has acted in different directions on the allele frequencies in different populations, leading to high differentiation.

### Within Continent Long-range Haplotype Test for Selection on CD Network

In addition to our F_ST_ based test, we utilized existing long-range haplotype data from the human genome diversity panel (HGDP) to examine patterns of selection across the CD background risk network. One way to approach the question of selection on a set of loci is to examine empirical observations in comparison to results from model-bound simulations. However, at this point in time, we are not comfortable specifying a population model due to the fact that traditional models of human demographic history are complicated by the presence of a substantial fraction of loci deriving from archaic human ancestors [35]–[37]. Rather, we chose to devise an empirical randomization outlier test. Specifically, we downloaded the European, East Asian, and Bantu HGDP tracks for normalized integrated haplotype score (iHS) from the UCSC genome table browser (genome.ucsc.edu). Using custom scripts, we extracted the highest iHS score from each CD region (Table S1) and calculated a mean network iHS score. We then used a randomization test to compare the CD network value to 100,000 randomly selected sets of independent loci. In selecting sets of random loci, we required all loci on the same chromosome to be at least two megabases (2 Mb) apart ensuring independence. We repeated this procedure for each continental region over a range of larger minimum distances to ensure consistency (data not shown). Some independently associated CD risk loci fall close enough to each other on the same chromosome to not be genetically independent. Therefore, we also repeated this test choosing only one such locus from each cluster of CD risk loci. In this implementation, we averaged the network iHS value over ten random draws before the randomization test (Figure 4). P-values were calculated as the fraction of 100,000 randomly generated networks that have a mean iHS value as high or higher than the CD network.

### Reduction of Heterozygosity in Ötzi from Low-coverage

To determine the effect of low coverage in the Ötzi genome on average heterozygosity across a small number of sites, we used the average weighted derived allele frequency in Europe (from 1000 Genomes Project) for each site to randomly generate a genotype for each site (repeated 500 times). For each genotype we randomly chose reads for each site according to the number of reads present at that site in the Ötzi genome (repeated 2500 times). This sampling procedure resulted in a set of 500 average heterozygosities expected based on allele frequency and 1,250,000 average heterozygosities based on allele frequency and random sampling of reads.

## Supporting Information

Table S1 **F_ST_ test results.** This table includes results for all loci included in each F_ST_ analysis. Loci are reported with positions from human genome build hg19 and are aligned with corresponding loci (hg18) from Trynka et al. 2011.
(XLSX)
